# Thyroid hormone and the developing hypothalamus

**DOI:** 10.3389/fnana.2015.00015

**Published:** 2015-02-20

**Authors:** Anneke Alkemade

**Affiliations:** Amsterdam Brain and Cognition Center, University of AmsterdamAmsterdam, Netherlands

**Keywords:** hypothalamus, brain development, thyroid hormone, paraventricular nucleus, deiodinase

## Abstract

Thyroid hormone (TH) plays an essential role in normal brain development and function. Both TH excess and insufficiency during development lead to structural brain abnormalities. Proper TH signaling is dependent on active transport of the prohormone thyroxine (T4) across the blood-brain-barrier and into brain cells. In the brain T4 undergoes local deiodination into the more active 3,3′,5-triiodothyronine (T3), which binds to nuclear TH receptors (TRs). TRs are already expressed during the first trimester of pregnancy, even before the fetal thyroid becomes functional. Throughout pregnancy, the fetus is largely dependent on the maternal TH supply. Recent studies in mice have shown that normal hypothalamic development requires intact TH signaling. In addition, the development of the human lateral hypothalamic zone coincides with a strong increase in T3 and TR mRNA concentrations in the brain. During this time the fetal hypothalamus already shows evidence for TH signaling. Expression of components crucial for central TH signaling show a specific developmental timing in the human hypothalamus. A coordinated expression of deiodinases in combination with TH transporters suggests that TH concentrations are regulated to prevent untimely maturation of brain cells. Even though the fetus depends on the maternal TH supply, there is evidence suggesting a role for the fetal hypothalamus in the regulation of TH serum concentrations. A decrease in expression of proteins involved in TH signaling towards the end of pregnancy may indicate a lower fetal TH demand. This may be relevant for the thyrotropin (TSH) surge that is usually observed after birth, and supports a role for the hypothalamus in the regulation of TH concentrations during the fetal period anticipating birth.

## Introduction

Thyroid hormone (TH) is crucial for normal fetal growth and maturation including the brain (Eayrs and Taylor, 1951; Morreale de Escobar et al., 1983; Legrand, 1984). Developmental TH deficiency impairs growth, and compromises adaptation to life outside the womb (Forhead et al., 1998; Hillman et al., 2012; Sferruzzi-Perri et al., 2013; Forhead and Fowden, 2014). The importance of TH during development is widely recognized and reflected in government interventions, such as iodine supplementation programs, as recommended by the World Health Organization (e.g., universal salt iodisation), and population wide screening for TH deficiency via the heel prick (Ford and LaFranchi, 2014). TH deficiency during development causes irreversible damage, which can largely be prevented by TH replacement therapy (Dubuis et al., 1996; Grüters and Krude, 2011).

During uterine development the fetus is largely dependent on the maternal TH supply, and TH receptors (TRs) are already present before the thyroid gland becomes functional (Morreale de Escobar et al., 2004, 2007). TH is implicated in many developmental processes in the brain including cell cycling, synaptogenesis, migration, plasticity, and myelination (Bernal, 2007). In both humans and rodents, brain development is not completed at, and continues after birth. From the moment of birth, the offspring no longer benefits from maternal TH supply. Insufficient TH availability, also during the postnatal developmental period, leads to severe neurological damage and mental retardation. TH insufficiency can be the result of iodine deficiency, or of defective formation of the thyroid gland (Deladoëy et al., 2007; Nilsson and Fagman, 2013; Szinnai, 2014). Interestingly, many studies have focused on the importance of TH in brain development, but much less is known about the development of the hypothalamic feedback loop of TH. When studying intrauterine development, it is not only the fetal hypothalamus that is in play, it is also that of the mother. In view of the cross-talk between systems it is often not possible to distinguish between the different (neuro-)endocrine systems in play. Rodent studies have been crucial for understanding TH signaling, but the research is complicated by interspecies differences that are observed between rodents, humans and other mammals. In the present review I will focus mainly on TH signaling in the developing human hypothalamus.

## TH signaling in the hypothalamus

**Figure 1:**
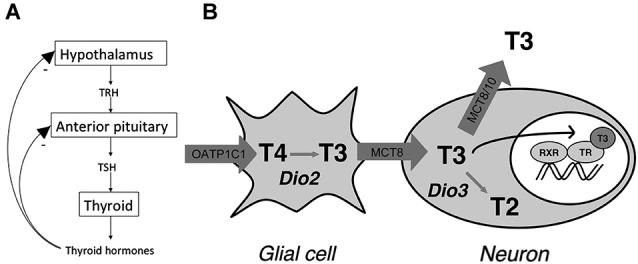
**(A)** Classic thyroid hormone (TH) feedback loop. TH exerts a negative feedback at the level of the hypothalamus and anterior pituitary. **(B)** Model for thyroid hormone (TH) transport and metabolism in the human hypothalamus. The model indicates distinct roles for the different TH transporters in hypothalamic TH action by affecting intracellular TH concentrations through import and efflux. T2 = 3,3′-T2 (Adapted from Alkemade et al., 2011).

Under normal conditions, TH signaling is controlled via a classic negative feedback pathway at the level of the anterior pituitary and hypothalamus (Figure 1A). Hypothalamic TH signaling is also involved in circadian rhythmicity, feeding and adaptation to environmental challenges (Costa-e-Sousa and Hollenberg, 2012). Interestingly, studies in anencephalic humans have shown that when the hypothalamus is not formed at all, pituitary-thyroid function still develops (Beck-Peccoz et al., 1992). Following normal brain development, hypophysiotropic neurons of the paraventricular nucleus (PVN) of the hypothalamus expressing thyrotropin releasing hormone (TRH) project to the portal system, via which it reaches the thyrotropin (TSH) producing cells of the anterior pituitary. TSH, which is subsequently released, binds to its receptor in the thyroid where TH is produced and released. TH is released predominantly as thyroxine (T4), a prohormone, and to a lesser extent as the active 3,3′,5-triiodothyronine (T3). T4 is converted locally into T3 providing a negative feedback at the level of the pituitary as well as the hypothalamus (Figure 1A). T3 mainly acts by regulation of gene expression via binding of nuclear TRs, although nongenomic effects of T3 have been described as well (Cheng et al., 2010; Davis et al., 2013).

A number of specific proteins are required for normal TH signaling in the brain including the hypothalamus. These proteins have been studied mainly in humans and rodents. TH cannot enter or exit the cell via passive diffusion and requires facilitated transport across the cell membrane, as well as the membrane of the cell nucleus (Visser et al., 2008; Heuer and Visser, 2009; Wirth et al., 2014). Proteins capable of transporting TH belong to a number of families including the sodium dependent organic anion tranporters (NTCP), organic anion transporting polypeptides (OATP), heterodimeric amino acid transporters (HAT) including light chains LAT1 and 2, and the monocarboxylate transporters MCT8 and 10 (Friesema et al., 1999, 2001; Hennemann et al., 2001; Roberts et al., 2008). The distribution patterns of these transporters throughout the body show significant overlap, and most of them are able to transport other molecules than iodothyronines as well. MCT8 appears to be specific for transporting iodothyronines (Friesema et al., 2003). OATP1C1 is expressed in endothelial cells of the blood brain barrier, as well as the choroid plexus (Roberts et al., 2008). MCT8 is also expressed in the human as well as the rodent choroid plexus (Heuer et al., 2005; Friesema et al., 2012). LAT1 and 2 do not appear to play any major role in the human brain (Wirth et al., 2014).

T4 is converted to T3 via intracellular outer ring deiodination. In the central nervous system conversion of T4 into T3 is mainly dependent on type 2 deiodinase (D2), and inactivation of T4 and T3 is dependent on inner ring deiodination by type 3 deiodinase (D3). Type 1 deiodinase (D1) does not appear to play a major role in rodent or human brain (Bianco et al., 2002). Interestingly, D2 is mainly expressed in glial cells of the hypothalamus. These include astrocytes and specialized glial cells called tanycytes, which are located at the ependymal layer of the third ventricle (Tu et al., 1997; Alkemade et al., 2005a). *Dio2 mRNA* expression in tanycytes shows a clear increase in response to hypothyroidism and iodine deficiency (Tu et al., 1997; Peeters et al., 2001).

In the cell nucleus, T3 binds the nuclear receptors encoded by the THRA and THRB genes, which give rise to TRα1, α2, β1 and β2 (Sap et al., 1986; Weinberger et al., 1986; Benbrook and Pfahl, 1987; König and Moura Neto, 2002). TRα2 does not bind T3, but exerts dominant negative effects on TRH transcription (Guissouma et al., 2014). Studies in knockout mice indicate that TRβ2 is crucial for the negative feedback loop controlling TH concentrations in the body (Abel et al., 2001). In addition, TRβ1 is also involved in regulating serum TSH (Guissouma et al., 2006). It is not entirely clear what the role for TRα in the negative feedback loop is. In the human and rat hypothalamus TRs are expressed in a number of nuclei, including the PVN where the hypophysiotropic TRH neurons are located (Lechan et al., 1994; Alkemade et al., 2005b). We, and others have proposed models for TH signaling in the hypothalamus, which involves both glial cells and neurons (Guadaño-Ferraz et al., 1997; Tu et al., 1997; Diano et al., 2003; Lechan and Fekete, 2004; Alkemade et al., 2005a; Figure 1B).

Hypothalamic TH signaling extends beyond the classical feedback loop affecting the TRH neurons of the PVN. In addition, functioning of other nuclear receptors such as the liver X receptor is dependent on thyroid status (Ghaddab-Zroud et al., 2014). We found in our previous studies on the human hypothalamus that the individual TR isoforms are expressed in a number of hypothalamic nuclei and studies in rats have shown comparable results. In addition, the supraoptic (SON), infundibular (IFN), tuberomamillary (TMN), and the lateral tuberal nucleus (NTL) express TRs. In rodents, TR isoforms have been described in the arcuate nucleus (ARC), the rodent equivalent of the IFN, as well as SON and PVN (Bradley et al., 1989; Cook et al., 1992; Lechan et al., 1994). The SON showed higher TRα than TRβ expression (Bradley et al., 1989). Studies in a transgenic mouse line with a GFP-labeled TRα1 (Wallis et al., 2010) have shown that the distribution of the TRα1 is even more widespread than we concluded from our studies on the human hypothalamus. In mice TRα1 was expressed in the majority of cerebral neurons, including many hypothalamic nuclei, as well as in tanycytes. Several explanations are possible for the discrepancy between our findings and findings in this transgenic mouse strain. It is possible that the antibodies we used for studying TR expression in the human hypothalamus lacked sensitivity to detect all TRs in the human hypothalamus, or, alternatively, interspecies differences may exist. Wallis et al. (2010) further showed TRα1 expression in nonneuronal cells including hypothalamic oligodendrocytes. In our earlier studies in humans we did not report on oligodendritic expression of TRs (Alkemade et al., 2005b). Interestingly, the absence of a functional MCT8 results a persistent hypomyelination in humans (López-Espíndola et al., 2014). The findings by Wallis et al. fit with the ubiquitous expression of the TH transporter OATP1C1 throughout the human hypothalamus, in both neurons as well as glial cells (Alkemade et al., 2011). In rodents Oatp1C1 does not show neuronal expression in the hypothalamus (Roberts et al., 2008). It is possible that other TH transporters are involved in the transport of TH in and out of TRα1 expressing neurons and glia in rodents. In addition, we have shown that MCT8 is expressed in both neurons and glial cells of the human hypothalamus. Interestingly, in humans MCT10 expression was confined to neurons in the majority of hypothalamic nuclei. The distinct distribution patterns support different roles for the individual TH transporters.

## TH signaling in the developing human hypothalamus

Very few studies have investigated the distribution of protein expression in the developing human hypothalamus, therefore, little is known about its chemoarchitecture. Human brain material obtained is not readily available, and tissue from embryo’s, fetuses and children is even scarcer. Koutcherov et al. (2002), studied 33 human hypothalamic specimens ranging in age from 9 weeks of gestation to 3 weeks after term birth. This study describes the development of human hypothalamic zones and individual nuclei and provides a clear schematic representation of the development of the human hypothalamus. Hypothalamic differentiation starts already during the first trimester of pregnancy. The lateral zone is the first zone to differentiate, and gives rise to the lateral hypothalamus (LH), Posterior hypothalamic area (PH), lateral tuberal hypothalamic nucleus (LTu) and perifornical hypothalamic nucleus (PeF). The core zone consists of a heterogeneous collection of nuclei positioned between the LH and midline structure, including medial preoptic nucleus (MPO), ventromedial nucleus (VMH), supramamillary nucleus (SUM) and mammillary bodies (Mb). The midline hypothalamus consists of structures differentiating in close proximity of the ventricular wall, including the suprachiasmatic nucleus (SCN), IFN, PVN and SON. These structures become evident during late gestation.

We have described the developmental expression of proteins involved in TH signaling in 15 fetal and infant human hypothalami obtained at various stages of development (Friesema et al., 2012). To my knowledge comparable studies investigating fetal functional neuroanatomy underlying TH signaling are not available for rodents. In our study on the developing human hypothalamus the first sampling point was at 17 weeks of gestation. Although we did not study TR expression in our human brain specimens, at this time both TRs and the ligand T3 have been reported in the human brain and TR mRNA increases strongly from 10–18 weeks gestation (Iskaros et al., 2000). Interestingly, during development TRα1 is not expressed in the ventricular zone in mice where neurons are born and proliferate (Wallis et al., 2010). These studies showed that TRα1 appears to be expressed in immature neurons, preceding the expression of NeuN. The TRα1 expression increases when cells reach their destination and differentiate. This means that TRα1 acts after cell cycle exit. The specific oligodendrocytic expression of TRα1 fits with data showing that oligodendrocyte precursors differentiate after T3 activation of a transiently expressed TR (Billon et al., 2002). This indicates that in mice TRα1 expression is required only during a specific time window in development (Wallis et al., 2010). Whether a similar pattern is also present in the (human) hypothalamus is unknown.

At 17 weeks of gestation the mamillary bodies become prominent and the LH, SON and IFN can already be distinguished, as well as the fornix and the anlage of the PVN (Koutcherov et al., 2002). In our human studies, we observed some MCT8 expression in blood vessels at 17 weeks, indicating possible presence of TH signaling (Friesema et al., 2012). We only observed few D3 positive neurons in the IFN, whereas D2 did not show any staining. These findings indicate T3 degradation, but not production in the human fetal hypothalamus at this moment in development. This is in line with the prevention of TH exposure during early stages of brain development, which may cause untimely maturation of brain cells as observed in rodents (Obregon et al., 2007). Interestingly, excess TH as a result of deletion of the *Dio3* gene in mice results in premature cerebellar differentiation, as well as a central hypothyroidism associated with defective TRH regulation, which persists throughout life (Hernandez et al., 2006, 2007; Peeters et al., 2013).

At 18 weeks gestation all T3 in the brain is produced via local deiodination (Ferreiro et al., 1988), which fits with D2 activity and T3 content in the cerebral cortex during the second trimester (Kester et al., 2004). At this time TH transporters are also expressed in the cerebral cortex (Chan et al., 2014). It does not appear that at this time point the human hypothalamus is capable of converting T4 into T3. This finding could reflect timing differences between distinct brain areas. At 18–23 weeks of gestation the PVN develops, and the PeF area as well as the LH become discernable. At the end of this period the SCN becomes visible (Koutcherov et al., 2002). At present no data are available on TH signaling at this time-point during development.

At 24–33 weeks the fetal human hypothalamus takes and adult like appearance. At 25 5/7 weeks, we found MCT8 expression in the PVN and IFN, which should now also express Neuropeptide Y (Koutcherov et al., 2002). In addition, MCT8 neurons showed a scattered pattern throughout the hypothalamus, as well as in tanycytes and blood vessels (Friesema et al., 2012). MCT10 showed expression in SON, PVN and IFN. D3 and OATP1C1 were also expressed in the IFN. At this time D2 was still very low and only found surrounding blood vessels. At 27 weeks MCT8 expression in PVN and LH persisted, and MCT10 showed more widespread expression in PVN, LH and SON. OATP1C1 was also present in LH, PVN and IFN. Hardly any D2 was observed, whereas the PVN showed prominent D3 expression.

At 27 2/7 weeks of gestation TH signaling appeared to increase. MCT8 was present in neurons of the PVN, IFN and LH, as well as in tanycytes. MCT10 was strongly expressed in the LH, as well as in neurons of the IFN, PVN and SON. OATP1C1 was present in blood vessels of the organum vasculosum laminae terminalis (OVLT) and in LH, IFN, PVN and SON neurons. D2 at this stage showed expression in tanycytes. In addition D2 staining surrounded blood vessels of the OVLT. Now D3 expression was not observed (Friesema et al., 2012). It is possible that the absence of D3 expression indicates an increased need for TH at this stage of development persisting to late gestation, after which TH signaling appears to decrease again. At 28 3/7 weeks gestation MCT8 staining was present in the IFN, PVN and LH. MCT10 and OATP1C1 also showed expression in the LH. In addition, OATP1C1 was also present in the IFN and in tanycytes, which also expressed D2. Again D3 showed very little expression.

From 34 weeks gestation term gestation, the NTL and TMN can develop further (Koutcherov et al., 2002). At 34 5/7 weeks the expression of all tested TH transporters was low. What is interesting to note, is that during late gestation (35 weeks), D2, MCT8 and OATP1C1 expression was very weak, and MCT10 expression was not observed. This was in contrast to the strong D3 expression observed in the IFN, TMN and LH. These observations led us to speculate that these observations may be related to the TSH surge observed in humans associated with birth, and that the hypothalamus may play a role in giving rise to this TSH surge. Although highly speculative, decreased T3 availability due to decreased outer ring deiodination and TH transport, together with increased inner ring deiodination by D3 could lead to upregulation of TRH, which is negatively regulated by T3. A very similar staining pattern was observed in hypothalami of children that were born at term. Inverse hypothalamic expression levels of D2 and 3 have also been observed in birds and mammals in the context of photoperiod setpoints (Watanabe et al., 2007).

**Figure 2:**
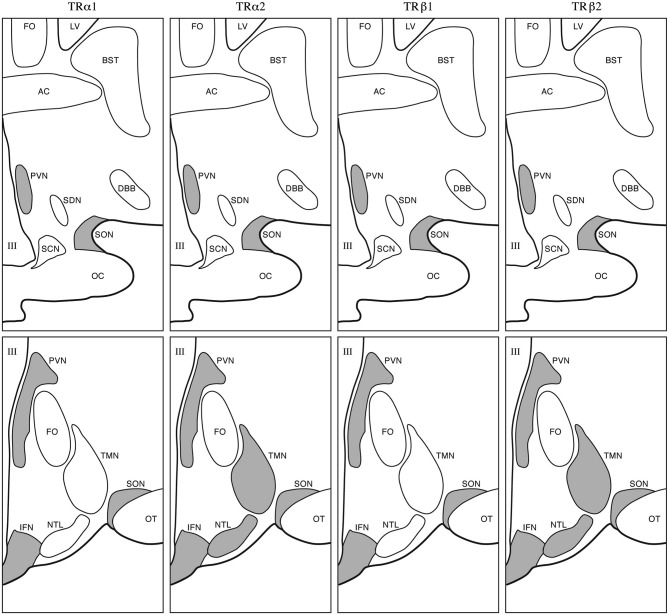
**Adult appearance of the human hypothalamus**. Gray areas indicate sites of TR isoform expression. Upper panels: rostral level; lower panels: caudal level. III, Third ventricle; AC, anterior commissure; BST, bed nucleus of the stria terminalis; DBB, diagonal band of Broca; FO, fornix; LV, lateral ventricle; NTL, nucleus tuberalis lateralis; OC, optic chiasm; OT, optic tract; SCN, suprachiasmatic nucleus; SDN, sexually dimorphic nucleus; SON, supraoptic nucleus; TMN, tuberomammilary nucleus (Taken from Alkemade et al., 2005b).

At *2 months of age* staining for MCT8 in SON, PVN, and in the lining of the third ventricle was found in humans. MCT10 immunoreactivity was present in SON and PVN. Sporadic expression was found in the LH. OATP1C1 showed a more scattered pattern and increased staining was observed in SON, PVN, TMN, IFN and the ependymal layer. D2 expression was present in blood vessels. D3 showed expression in PVN as well as other hypothalamic nuclei. In children *aged 20–29 months of age*, we found moderate MCT8 and moderate to strong MCT10 staining in PVN, SON, IFN and weak staining in the LH. OATP1C1 was present in PVN, SON, IFN and in some cells in the ependymal layer of the third ventricle. In addition, scattered OATP1C1 positive cells were present, as well as staining in NTL, TMN, SCN and LH. D2 was expressed in blood vessels especially in the IFN and in tanycytes. At this time point stainings very much resembled our observations in the adult hypothalamus (Alkemade et al., 2005a, 2011; Figure 2).

**Figure 3:**
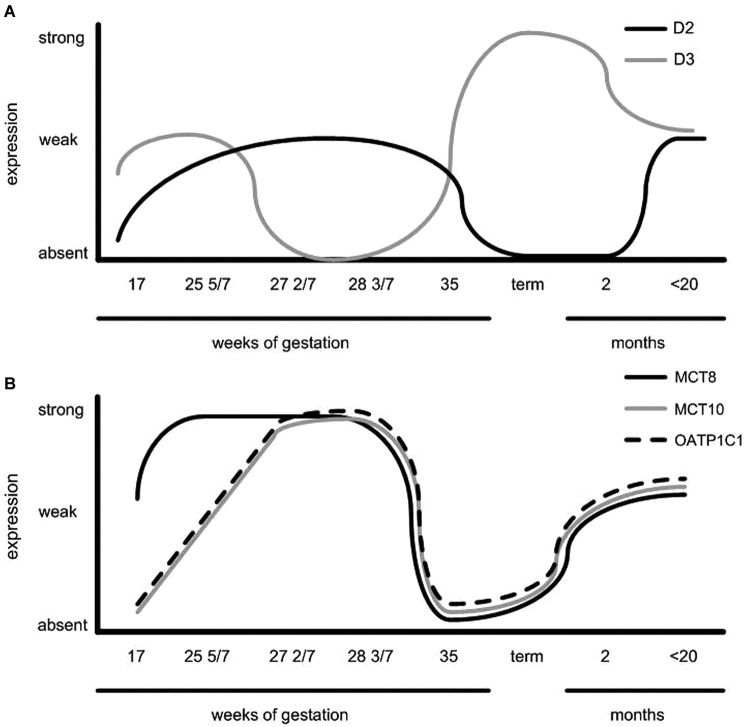
**Schematic representation of (A) D2 expression in the human infundibular nucleus/median eminence and periventricular area, D3 expression in the paraventricular nucleus (PVN) and (B) TH transporter expression throughout PVN development**. Note the opposite expression pattern of D3 vs. TH transporters and D2 expression (Taken from Friesema et al., 2012).

Hypothalamic expression patterns throughout the developmental period studied are summarized in Figure 3. This figure indicates that regulation of proteins involved in TH signaling may play an important role in timing of TH availability at different stages of development.

## Defective hypothalamic TH signaling

Defects in proteins involved in hypothalamic TH signaling are potentially reflected in alterations in serum TH and TSH concentrations. A number of mutations in, TRs and TH transporters has been described. These syndromes are described excellently in a recent review (Dumitrescu and Refetoff, 2013), and here only findings relevant for HPT-axis regulation and the brain are recapitulated.

### TRs

Complete deletion of all TRs in mice causes serum TSH levels that are 500-fold higher than those of the WT mice, and T4 concentrations 12-fold above the average normal mean (Gauthier et al., 1999). In humans both mutations in the THRA and THRB genes have been reported.

### Resistance to THα

The first subject described suffering from Resistance to THα (RTHα), was diagnosed only recently (Bochukova et al., 2012). RTHα is caused by a mutation in the *THRA* gene, which encodes TRα1 and α2. The mutation caused a truncation of the protein, which lacked the C-terminal α-helix. This 6-year old girl showed low-normal or subnormal levels of total T4 and free T4, high-normal or elevated levels of total T3 and free T3, in addition to normal levels of TSH (Bochukova et al., 2012). Since the description of this young girl, additional patients with RTHα have been described in literature. RTHα patients show cognitive impairment and macrocephaly (Bochukova et al., 2012; van Mullem et al., 2013). In addition, the phenotype observed in a patient with mutations both in TRα1 and α2 was very similar (Moran et al., 2014). Serum TH concentrations do not suggest any major impairment of the classic hypothalamic feedback system. This is corroborated by findings in mice. Mouse models with a mutant TRα1, show a range of phenotypes, dependent on the specific mutation or deletion. Brain changes have been described as well (Wallis et al., 2008; Mittag et al., 2010), and changes resulting from defects in the autonomic nervous system including impaired cardiovascular response to stress, activity and environmental temperature changes. The hypothalamus has only been studied in detail in one of these mouse models, showing the absence of a parvalbumin positive neuronal population in the anterior hypothalamic area with consequences for the regulation of the cardiovascular system (Mittag et al., 2013). The HPA-axis was not affected in these mutant mice as assessed by hypothalamic corticotropin releasing hormone (CRH) and pro-opiomelanocortin (POMC) expression (Mittag et al., 2010).

### Resistance to THβ

Patients harboring mutations in the TRβ1 show more pronounced changes in serum TH concentrations. For the THRB gene number of mutation hotspots have been defined (Dumitrescu and Refetoff, 2013). Characteristic are elevated freeT4 levels, and to a lesser extent T3. In addition, TSH is normal or slightly increased, and responsive to TRH. Patients generally do not display the usual metabolic symptoms associated with hyperthyroidism, although they often present with goiter (Refetoff et al., 1993). TRβ2 knockout mice show a severe disruption of the hypothalamic TH feedback system (Abel et al., 2001).

### TH transporters

In MCT8 deficient subjects serum TSH is modestly increased, which fits with the decreased serum T_4_ concentration but not with the elevated serum T_3_ level. Since MCT8 is expressed in the hypothalamus and pituitary, it is likely that inactivation of the gene interferes with the negative feedback at the level of the hypothalamus (Fliers et al., 2006). In addition, defects in brain development have been described in patients lacking MCT8 (López-Espíndola et al., 2014). Rodent models for MCT8 mutations do not reproduce the severe psychomotor phenotype observed in humans, but do faithfully reproduce the biochemical phenotype (Trajkovic et al., 2007). In *Mct8*KO mice, hypothalamic TRH expression is markedly increased and high T_3_ doses are needed to suppress it. In humans there is clear expression of MCT10 and OATP1C1 already in the second trimester of pregnancy. The presence of these transporters is does compensate for the absence of MCT8 as evidenced by the neurological defects observed in patients lacking MCT8. Wirth et al. suggest that Lat2 might compensate for the Mct8 deletion in mice. This is unlikely in humans, since developing neurons in the human brain only show very low LAT2 expression (Wirth et al., 2009). It is possible that in rodents other transporter variants such as Oatp1a4 and Oatp1a5 may further compensate for MCT8 defects. These transporters appear not to have orthologs in the human brain (Suzuki and Abe, 2008).

### TH conversion defects

Selenocysteine insertion sequence (SECIS) binding protein 2 (SBP2) plays an important role in insertion of selenocysteine into selenoproteins such as deidonases. Defects in the *SBP2* gene therefore interfere with the production of these conversion enzymes, resulting in high T4 and rT3, low T3, normal or slightly elevated TSH concentrations (Dumitrescu et al., 2005). Genetic variants in *DIO2* in humans do not show any clear phenotypic changes (Zevenbergen et al., 2014).

## Conclusion

TH signaling involves facilitated transport, local conversion and receptor binding. TH signaling is therefore dependent on a number of proteins, each of which can become defective, thereby affecting a plethora of processes modulated by TH. The effects of mutations in TH signaling on the functional neuroanatomy, which underlies the classic negative feedback of TH on the hypothalamus is dependent on the affected gene, the type of mutation, as well as the compensatory mechanisms, which appear to differ between species. These effects are largely assessed by evaluation of biochemical parameters. Detailed studies on the functional neuroanatomy underlying hypothalamic TH signaling are scarce, and are complicated by interspecies differences, as well as the limited availability of human postmortem brain material for research purposes. Future studies on the expression of TR isoforms in the developing (human) hypothalamus would strongly improve our understanding of central TH signaling during development.

## Conflict of interest statement

The author declares that the research was conducted in the absence of any commercial or financial relationships that could be construed as a potential conflict of interest.

## References

[B1] Abel E. D. Ahima R. S. Boers M. E. Elmquist J. K. Wondisford F. E. (2001). Critical role for thyroid hormone receptor β2 in the regulation of paraventricular thyrotropin-releasing hormone neurons. J. Clin. Invest. 107, 1017–1023. 10.1172/jci10858 11306605 PMC199552

[B2] Alkemade A. Friesema E. C. Kalsbeek A. Swaab D. F. Visser T. J. Fliers E. (2011). Expression of thyroid hormone transporters in the human hypothalamus. J. Clin. Endocrinol. Metab. 96, E967–E971. 10.1210/jc.2010-2750 21508134

[B3] Alkemade A. Friesema E. C. Unmehopa U. A. Fabriek B. O. Kuiper G. G. Leonard J. L.. (2005a). Neuroanatomical pathways for thyroid hormone feedback in the human hypothalamus. J. Clin. Endocrinol. Metab. 90, 4322–4334. 10.1210/jc.2004-2567 15840737

[B4] Alkemade A. Vuijst C. L. Unmehopa U. A. Bakker O. Vennström B. Wiersinga W. M.. (2005b). Thyroid hormone receptor expression in the human hypothalamus and anterior pituitary. J. Clin. Endocrinol. Metab. 90, 904–912. 10.1210/jc.2004-0474 15562027

[B5] Beck-Peccoz P. Cortelazzi D. Persani L. Papandreou M. J. Asteria C. Borgato S.. (1992). Maturation of pituitary-thyroid function in the anencephalic fetus. Acta Med. Austriaca 19(Suppl. 1), 72–76. 1519459

[B6] Benbrook D. Pfahl M. (1987). A novel thyroid hormone receptor encoded by a cDNA clone from a human testis library. Science 238, 788–791. 10.1126/science.3672126 3672126

[B7] Bernal J. (2007). Thyroid hormone receptors in brain development and function. Nat. Clin. Pract. Endocrinol. Metab. 3, 249–259. 10.1038/ncpendmet0424 17315033

[B8] Bianco A. C. Salvatore D. Gereben B. Berry M. J. Larsen P. R. (2002). Biochemistry, cellular and molecular biology and physiological roles of the iodothyronine selenodeiodinases. Endocr. Rev. 23, 38–89. 10.1210/er.23.1.38 11844744

[B9] Billon N. Jolicoeur C. Tokumoto Y. Vennström B. Raff M. (2002). Normal timing of oligodendrocyte development depends on thyroid hormone receptor alpha 1 (TRα1). EMBO J. 21, 6452–6460. 10.1093/emboj/cdf662 12456652 PMC136965

[B10] Bochukova E. Schoenmakers N. Agostini M. Schoenmakers E. Rajanayagam O. Keogh J. M.. (2012). A mutation in the thyroid hormone receptor alpha gene. N. Engl. J. Med. 366, 243–249. 10.1056/NEJMoa1110296 22168587

[B11] Bradley D. J. Young W. S. 3rd Weinberger C. (1989). Differential expression of alpha and beta thyroid hormone receptor genes in rat brain and pituitary. Proc. Natl. Acad. Sci. U S A 86, 7250–7254. 10.1073/pnas.86.18.7250 2780568 PMC298035

[B12] Chan S. Y. Hancox L. A. Martín-Santos A. Loubière L. S. Walter M. N. González A. M.. (2014). MCT8 expression in human fetal cerebral cortex is reduced in severe intrauterine growth restriction. J. Endocrinol. 220, 85–95. 10.1530/JOE-13-0400 24204008 PMC3921694

[B13] Cheng S. Y. Leonard J. L. Davis P. J. (2010). Molecular aspects of thyroid hormone actions. Endocr. Rev. 31, 139–170. 10.1210/er.2009-0007 20051527 PMC2852208

[B14] Cook C. B. Kakucska I. Lechan R. M. Koenig R. J. (1992). Expression of thyroid hormone receptor beta 2 in rat hypothalamus. Endocrinology 130, 1077–1079. 10.1210/en.130.2.1077 1733708

[B15] Costa-e-Sousa R. H. Hollenberg A. N. (2012). Minireview: the neural regulation of the hypothalamic-pituitary-thyroid axis. Endocrinology 153, 4128–4135. 10.1210/en.2012-1467 22759379 PMC3423621

[B16] Davis P. J. Lin H. Y. Tang H. Y. Davis F. B. Mousa S. A. (2013). Adjunctive input to the nuclear thyroid hormone receptor from the cell surface receptor for the hormone. Thyroid 23, 1503–1509. 10.1089/thy.2013.0280 24011085

[B17] Deladoëy J. Vassart G. Van Vliet G. (2007). Possible non-Mendelian mechanisms of thyroid dysgenesis. Endocr. Dev. 10, 29–42. 10.1159/000106818 17684388

[B18] Diano S. Leonard J. L. Meli R. Esposito E. Schiavo L. (2003). Hypothalamic type II iodothyronine deiodinase: a light and electron microscopic study. Brain Res. 976, 130–134. 10.1016/s0006-8993(03)02692-1 12763631

[B19] Dubuis J. M. Glorieux J. Richer F. Deal C. L. Dussault J. H. Van Vliet G. (1996). Outcome of severe congenital hypothyroidism: closing the developmental gap with early high dose levothyroxine treatment. J. Clin. Endocrinol. Metab. 81, 222–227. 10.1210/jcem.81.1.8550756 8550756

[B20] Dumitrescu A. M. Liao X. H. Abdullah M. S. Lado-Abeal J. Majed F. A. Moeller L. C.. (2005). Mutations in SECISBP2 result in abnormal thyroid hormone metabolism. Nat. Genet. 37, 1247–1252. 10.1038/ng1654 16228000

[B21] Dumitrescu A. M. Refetoff S. (2013). The syndromes of reduced sensitivity to thyroid hormone. Biochim. Biophys. Acta 1830, 3987–4003. 10.1016/j.bbagen.2012.08.005 22986150 PMC3528849

[B22] Eayrs J. T. Taylor S. H. (1951). The effecot of thyroid deficiency induced by methylthiouracil on the maturation of the central nervous system. J. Anat. 85, 350–358. 14888602 PMC1273415

[B23] Ferreiro B. Bernal J. Goodyer C. G. Branchard C. L. (1988). Estimation of nuclear thyroid hormone receptor saturation in human fetal brain and lung during early gestation. J. Clin. Endocrinol. Metab. 67, 853–856. 10.1210/jcem-67-4-853 3417852

[B24] Fliers E. Unmehopa U. A. Alkemade A. (2006). Functional neuroanatomy of thyroid hormone feedback in the human hypothalamus and pituitary gland. Mol. Cell. Endocrinol. 251, 1–8. 10.1016/j.mce.2006.03.042 16707210

[B25] Ford G. LaFranchi S. H. (2014). Screening for congenital hypothyroidism: a worldwide view of strategies. Best Pract. Res. Clin. Endocrinol. Metab. 28, 175–187. 10.1016/j.beem.2013.05.008 24629860

[B26] Forhead A. J. Fowden A. L. (2014). Thyroid hormones in fetal growth and prepartum maturation. J. Endocrinol. 221, R87–R103. 10.1530/JOE-14-0025 24648121

[B27] Forhead A. J. Li J. Gilmour R. S. Fowden A. L. (1998). Control of hepatic insulin-like growth factor II gene expression by thyroid hormones in fetal sheep near term. Am. J. Physiol. 275, E149–E156. 9688886 10.1152/ajpendo.1998.275.1.E149

[B28] Friesema E. C. Docter R. Moerings E. P. Stieger B. Hagenbuch B. Meier P. J.. (1999). Identification of thyroid hormone transporters. Biochem. Biophys. Res. Commun. 254, 497–501. 10.1006/bbrc.1998.9974 9918867

[B29] Friesema E. C. Docter R. Moerings E. P. Verrey F. Krenning E. P. Hennemann G.. (2001). Thyroid hormone transport by the heterodimeric human system L amino acid transporter. Endocrinology 142, 4339–4348. 10.1210/en.142.10.4339 11564694

[B30] Friesema E. C. Ganguly S. Abdalla A. Manning Fox J. E. Halestrap A. P. Visser T. J. (2003). Identification of monocarboxylate transporter 8 as a specific thyroid hormone transporter. J. Biol. Chem. 278, 40128–40135. 10.1074/jbc.m300909200 12871948

[B31] Friesema E. C. Visser T. J. Borgers A. J. Kalsbeek A. Swaab D. F. Fliers E.. (2012). Thyroid hormone transporters and deiodinases in the developing human hypothalamus. Eur. J. Endocrinol. 167, 379–386. 10.1530/EJE-12-0177 22723621

[B32] Gauthier K. Chassande O. Plateroti M. Roux J. P. Legrand C. Pain B.. (1999). Different functions for the thyroid hormone receptors TR*α* and TR*β* in the control of thyroid hormone production and post-natal development. EMBO J. 18, 623–631. 10.1093/emboj/18.3.623 9927422 PMC1171155

[B33] Ghaddab-Zroud R. Seugnet I. Steffensen K. R. Demeneix B. A. Clerget-Froidevaux M. S. (2014). Liver X receptor regulation of thyrotropin-releasing hormone transcription in mouse hypothalamus is dependent on thyroid status. PLoS One 9:e106983. 10.1371/journal.pone.0106983 25229406 PMC4167690

[B34] Grüters A. Krude H. (2011). Detection and treatment of congenital hypothyroidism. Nat. Rev. Endocrinol. 8, 104–113. 10.1038/nrendo.2011.160 22009163

[B35] Guadaño-Ferraz A. Obregón M. J. St Germain D. L. Bernal J. (1997). The type 2 iodothyronine deiodinase is expressed primarily in glial cells in the neonatal rat brain. Proc. Natl. Acad. Sci. U S A 94, 10391–10396. 10.1073/pnas.94.19.10391 9294221 PMC23373

[B36] Guissouma H. Froidevaux M. S. Hassani Z. Demeneix B. A. (2006). In vivo siRNA delivery to the mouse hypothalamus confirms distinct roles of TR beta isoforms in regulating TRH transcription. Neurosci. Lett. 406, 240–243. 10.1016/j.neulet.2006.07.041 16930836

[B37] Guissouma H. Ghaddab-Zroud R. Seugnet I. Decherf S. Demeneix B. Clerget-Froidevaux M. S. (2014). TR alpha 2 exerts dominant negative effects on hypothalamic Trh transcription in vivo. PLoS One 9:e95064. 10.1371/journal.pone.0095064 24747825 PMC3991681

[B38] Hennemann G. Docter R. Friesema E. C. de Jong M. Krenning E. P. Visser T. J. (2001). Plasma membrane transport of thyroid hormones and its role in thyroid hormone metabolism and bioavailability. Endocr. Rev. 22, 451–476. 10.1210/er.22.4.451 11493579

[B39] Hernandez A. Martinez M. E. Fiering S. Galton V. A. St Germain D. (2006). Type 3 deiodinase is critical for the maturation and function of the thyroid axis. J. Clin. Invest. 116, 476–484. 10.1172/jci26240 16410833 PMC1326144

[B40] Hernandez A. Martinez M. E. Liao X. H. Van Sande J. Refetoff S. Galton V. A.. (2007). Type 3 deiodinase deficiency results in functional abnormalities at multiple levels of the thyroid axis. Endocrinology 148, 5680–5687. 10.1210/en.2007-0652 17823249

[B41] Heuer H. Maier M. K. Iden S. Mittag J. Friesema E. C. Visser T. J.. (2005). The monocarboxylate transporter 8 linked to human psychomotor retardation is highly expressed in thyroid hormone-sensitive neuron populations. Endocrinology 146, 1701–1706. 10.1210/en.2004-1179 15661862

[B42] Heuer H. Visser T. J. (2009). Minireview: pathophysiological importance of thyroid hormone transporters. Endocrinology 150, 1078–1083. 10.1210/en.2008-1518 19179441

[B43] Hillman N. H. Kallapur S. G. Jobe A. H. (2012). Physiology of transition from intrauterine to extrauterine life. Clin. Perinatol. 39, 769–783. 10.1016/j.clp.2012.09.009 23164177 PMC3504352

[B44] Iskaros J. Pickard M. Evans I. Sinha A. Hardiman P. Ekins R. (2000). Thyroid hormone receptor gene expression in first trimester human fetal brain. J. Clin. Endocrinol. Metab. 85, 2620–2623. 10.1210/jc.85.7.2620 10902817

[B45] Kester M. H. Martinez de Mena R. Obregon M. J. Marinkovic D. Howatson A. Visser T. J.. (2004). Iodothyronine levels in the human developing brain: major regulatory roles of iodothyronine deiodinases in different areas. J. Clin. Endocrinol. Metab. 89, 3117–3128. 10.1210/jc.2003-031832 15240580

[B46] König S. Moura Neto V. (2002). Thyroid hormone actions on neural cells. Cell. Mol. Neurobiol. 22, 517–544. 10.1023/A:1021828218454 12585678 PMC11533805

[B47] Koutcherov Y. Mai J. K. Ashwell K. W. Paxinos G. (2002). Organization of human hypothalamus in fetal development. J. Comp. Neurol. 446, 301–324. 10.1002/cne.10175 11954031

[B48] Lechan R. M. Fekete C. (2004). Feedback regulation of thyrotropin-releasing hormone (TRH): mechanisms for the non-thyroidal illness syndrome. J. Endocrinol. Invest. 27, 105–119. 15481810

[B49] Lechan R. M. Qi Y. Jackson I. M. Mahdavi V. (1994). Identification of thyroid hormone receptor isoforms in thyrotropin-releasing hormone neurons of the hypothalamic paraventricular nucleus. Endocrinology 135, 92–100. 10.1210/en.135.1.92 7516871

[B50] Legrand J. (1984). “Effects of thyroid hormones on central nervous system,” in Neurobehavioral Teratology, ed Yanai J. (Amsterdam: Elsevier), 331–363.

[B51] López-Espíndola D. Morales-Bastos C. Grijota-Martínez C. Liao X. H. Lev D. Sugo E.. (2014). Mutations of the thyroid hormone transporter MCT8 cause prenatal brain damage and persistent hypomyelination. J. Clin. Endocrinol. Metab. 99, E2799–E2804. 10.1210/jc.2014-2162 25222753 PMC4255116

[B52] Mittag J. Davis B. Vujovic M. Arner A. Vennström B. (2010). Adaptations of the autonomous nervous system controlling heart rate are impaired by a mutant thyroid hormone receptor-α1. Endocrinology 151, 2388–2395. 10.1210/en.2009-1201 20228172

[B53] Mittag J. Lyons D. J. Sallstrom J. Vujovic M. Dudazy-Gralla S. Warner A.. (2013). Thyroid hormone is required for hypothalamic neurons regulating cardiovascular functions. J. Clin. Invest. 123, 509–516. 10.1172/JCI65252 23257356 PMC3533300

[B54] Moran C. Agostini M. Visser W. E. Schoenmakers E. Schoenmakers N. Offiah A. C.. (2014). Resistance to thyroid hormone caused by a mutation in thyroid hormone receptor (TR)α1 and TRα: clinical, biochemical and genetic analyses of three related patients. Lancet Diabetes Endocrinol. 2, 619–626. 10.1016/s2213-8587(14)70111-1 24969835 PMC5989926

[B55] Morreale de Escobar G. Escobar del Rey F. Ruiz-Marcos A. (1983). “Thyroid hormone and the developing brain,” in Congenital Hypothyroidism, eds Dussault J. H. Walker P. (New York: MArcel Decker Inc.), 85–126.

[B56] Morreale de Escobar G. Obregón M. J. Escobar del Rey F. (2004). Maternal thyroid hormones early in pregnancy and fetal brain development. Best Pract. Res. Clin. Endocrinol. Metab. 18, 225–248. 10.1016/s1521-690x(04)00022-3 15157838

[B57] Morreale de Escobar G. Obregón M. J. Escobar del Rey F. (2007). Iodine deficiency and brain development in the first half of pregnancy. Public Health Nutr. 10, 1554–1570. 10.1017/S1368980007360928 18053280

[B58] Nilsson M. Fagman H. (2013). Mechanisms of thyroid development and dysgenesis: an analysis based on developmental stages and concurrent embryonic anatomy. Curr. Top. Dev. Biol. 106, 123–170. 10.1016/B978-0-12-416021-7.00004-3 24290349

[B59] Obregon M. J. Calvo R. M. Escobar del Rey F. Morreale de Escobar G. (2007). Ontogenesis of thyroid function and interactions with maternal function. Endocr. Dev. 10, 86–98. 10.1159/000106821 17684391

[B60] Peeters R. Fekete C. Goncalves C. Legradi G. Tu H. M. Harney J. W.. (2001). Regional physiological adaptation of the central nervous system deiodinases to iodine deficiency. Am. J. Physiol. Endocrinol. Metab. 281, E54–E61. 11404222 10.1152/ajpendo.2001.281.1.E54

[B61] Peeters R. P. Hernandez A. Ng L. Ma M. Sharlin D. S. Pandey M.. (2013). Cerebellar abnormalities in mice lacking type 3 deiodinase and partial reversal of phenotype by deletion of thyroid hormone receptor α1. Endocrinology 154, 550–561. 10.1210/en.2012-1738 23161871 PMC3529370

[B62] Refetoff S. Weiss R. E. Usala S. J. (1993). The syndromes of resistance to thyroid hormone. Endocr. Rev. 14, 348–399. 10.1210/er.14.3.348 8319599

[B63] Roberts L. M. Woodford K. Zhou M. Black D. S. Haggerty J. E. Tate E. H.. (2008). Expression of the thyroid hormone transporters monocarboxylate transporter-8 (SLC16A2) and organic ion transporter-14 (SLCO1C1) at the blood-brain barrier. Endocrinology 149, 6251–6261. 10.1210/en.2008-0378 18687783

[B64] Sap J. Munoz A. Damm K. Goldberg Y. Ghysdael J. Leutz A.. (1986). The c-erb-A protein is a high-affinity receptor for thyroid hormone. Nature 324, 635–640. 10.1038/324635a0 2879242

[B65] Sferruzzi-Perri A. N. Vaughan O. R. Forhead A. J. Fowden A. L. (2013). Hormonal and nutritional drivers of intrauterine growth. Curr. Opin. Clin. Nutr. Metab. Care 16, 298–309. 10.1097/MCO.0b013e32835e3643 23340010

[B66] Suzuki T. Abe T. (2008). Thyroid hormone transporters in the brain. Cerebellum 7, 75–83. 10.1007/s12311-008-0029-9 18418673

[B67] Szinnai G. (2014). Clinical genetics of congenital hypothyroidism. Endocr. Dev. 26, 60–78. 10.1159/000363156 25231445

[B68] Trajkovic M. Visser T. J. Mittag J. Horn S. Lukas J. Darras V. M.. (2007). Abnormal thyroid hormone metabolism in mice lacking the monocarboxylate transporter 8. J. Clin. Invest. 117, 627–635. 10.1172/JCI28253 17318265 PMC1797602

[B69] Tu H. M. Kim S. W. Salvatore D. Bartha T. Legradi G. Larsen P. R.. (1997). Regional distribution of type 2 thyroxine deiodinase messenger ribonucleic acid in rat hypothalamus and pituitary and its regulation by thyroid hormone. Endocrinology 138, 3359–3368. 10.1210/en.138.8.3359 9231788

[B70] van Mullem A. A. Chrysis D. Eythimiadou A. Chroni E. Tsatsoulis A. de Rijke Y. B.. (2013). Clinical phenotype of a new type of thyroid hormone resistance caused by a mutation of the TRα1 receptor: consequences of LT4 treatment. J. Clin. Endocrinol. Metab. 98, 3029–3038. 10.1210/jc.2013-1050 23633213

[B71] Visser W. E. Friesema E. C. Jansen J. Visser T. J. (2008). Thyroid hormone transport in and out of cells. Trends Endocrinol. Metab. 19, 50–56. 10.1016/j.tem.2007.11.003 18291666

[B72] Wallis K. Dudazy S. van Hogerlinden M. Nordström K. Mittag J. Vennström B. (2010). The thyroid hormone receptor α1 protein is expressed in embryonic postmitotic neurons and persists in most adult neurons. Mol. Endocrinol. 24, 1904–1916. 10.1210/me.2010-0175 20739404 PMC5417394

[B73] Wallis K. Sjögren M. van Hogerlinden M. Silberberg G. Fisahn A. Nordström K.. (2008). Locomotor deficiencies and aberrant development of subtype-specific GABAergic interneurons caused by an unliganded thyroid hormone receptor α1. J. Neurosci. 28, 1904–1915. 10.1523/JNEUROSCI.5163-07.2008 18287507 PMC6671444

[B74] Watanabe T. Yamamura T. Watanabe M. Yasuo S. Nakao N. Dawson A.. (2007). Hypothalamic expression of thyroid hormone-activating and -inactivating enzyme genes in relation to photorefractoriness in birds and mammals. Am. J. Physiol. Regul. Integr. Comp. Physiol. 292, R568–R572. 10.1152/ajpregu.00521.2006 17197645

[B75] Weinberger C. Thompson C. C. Ong E. S. Lebo R. Gruol D. J. Evans R. M. (1986). The c-erb-A gene encodes a thyroid hormone receptor. Nature 324, 641–646. 10.1038/324641a0 2879243

[B76] Wirth E. K. Roth S. Blechschmidt C. Hölter S. M. Becker L. Racz I.. (2009). Neuronal 3′,3,5-triiodothyronine (T3) uptake and behavioral phenotype of mice deficient in Mct8, the neuronal T3 transporter mutated in Allan-Herndon-Dudley syndrome. J. Neurosci. 29, 9439–9449. 10.1523/JNEUROSCI.6055-08.2009 19641107 PMC6666526

[B77] Wirth E. K. Schweizer U. Köhrle J. (2014). Transport of thyroid hormone in brain. Front. Endocrinol. (Lausanne) 5:98. 10.3389/fendo.2014.00098 25009532 PMC4067591

[B78] Zevenbergen C. Klootwijk W. Peeters R. P. Medici M. de Rijke Y. B. Huisman S. A.. (2014). Functional analysis of novel genetic variation in the thyroid hormone activating type 2 deiodinase. J. Clin. Endocrinol. Metab. 99, E2429–E2436. 10.1210/jc.2014-2281 25140401

